# A prediction model for mortality risk in melioidosis patients based on clinical and laboratory indicators: A single-center retrospective study

**DOI:** 10.1371/journal.pntd.0014516

**Published:** 2026-07-14

**Authors:** Huimin Zhao, Pei Zhang, Shijia Li, Xuehan Duan, Hua Wu

**Affiliations:** 1 Department of Clinical Laboratory, Hainan Women and Children’s Medical Center (Hainan Medical University School of Pediatrics), Haikou, China; 2 Hainan Medical University, Haikou, China; 3 Department of Laboratory Medicine, Hainan General Hospital, Hainan Affiliated Hospital of Hainan Medical University, Xiuying, Haikou, Hainan, China; 4 Department of Blood Transfusion, Hainan General Hospital, Hainan Affiliated Hospital of Hainan Medical University, Xiuying, Haikou, Hainan, China; University of Texas Medical Branch, UNITED STATES OF AMERICA

## Abstract

Melioidosis, caused by *Burkholderia pseudomallei*, carries a high mortality rate, particularly in acute severe cases. This study aimed to develop a practical prediction model for in-hospital mortality using routinely available clinical data. We retrospectively analyzed 283 melioidosis patients from Hainan General Hospital (2010–2024). Compared to survivors, non-survivors had significantly higher neutrophil percentage, C-reactive protein (CRP), and urea nitrogen levels, and lower platelet counts (PLT) (all *P* < 0.05) based on the last available measurements during hospitalization. Multivariate logistic regression identified these four variables as independent risk factors for mortality. A combined model integrating neutrophil percentage, PLT, CRP, and urea nitrogen demonstrated outstanding discriminative performance, with an area under the curve (AUC) of 0.957 (95% CI: 0.925–0.989), sensitivity of 94.9%, and specificity of 86.4%, significantly outperforming any single indicator. This high-accuracy model, based on inexpensive and universally available parameters, shows great potential for risk assessment during hospitalization of melioidosis patients in resource-limited endemic settings, facilitating timely intensive intervention.

## Author summary

In this study, we developed a simple tool to predict which melioidosis patients are at the highest risk of dying in the hospital. By analyzing data from 283 patients, we found that a high percentage of neutrophils (a type of white blood cell), a low platelet count, and high levels of two common blood tests (CRP and urea nitrogen) were strongly linked to death. These four easy-to-obtain measures form the basis of our prediction tool. They were taken from the last available tests during the patient’s hospital stay (just before death for non-survivors or before discharge for survivors). These values capture the most severe physiological state during the patient’s clinical course, reflecting cumulative deterioration before death or recovery. Combining these four measures into a single model allowed us to identify high-risk patients with a sensitivity of 94.9% and a specificity of 86.4%. This tool is particularly valuable for hospitals in areas where melioidosis is common but resources are limited, as it uses only standard tests and could be built into patient record systems to alert doctors to high-risk patients prompting closer monitoring and more aggressive treatment to save lives.

## Introduction

Melioidosis is a potentially fatal zoonotic disease caused by the gram-negative bacterium *Burkholderia pseudomallei* [1]. Endemic in tropical and subtropical regions, its clinical presentation ranges from localized abscess to fulminant sepsis and multi-organ failure, resulting in high mortality rates that exceed 40% in septic shock [1,2]. Epidemiological studies from endemic foci like Southeast Asia and northern Australia consistently report a significant male predominance among cases [3,4].

Diabetes mellitus is a well-established major risk factor for acquiring melioidosis across Asia [5,6]. However, in the context of acute, severe infection requiring hospitalization, the independent prognostic impact of diabetes may be attenuated or overshadowed by the overwhelming severity of the acute infectious process itself [7,8]. This highlights the critical need for tools that can rapidly assess acute disease severity in hospitalized patients.

Currently, early and accurate prognostic stratification in melioidosis remains challenging. Although prior studies have explored associations between various demographic, comorbidity, and laboratory factors with outcomes, many rely on univariate analyses or report inconsistent findings [9]. Previous studies have proposed scoring systems for predicting mortality in melioidosis [10,11]. There is a lack of validated, practical prediction models that utilize routinely available clinical data. Therefore, this single-center retrospective study aimed to systematically identify independent risk factors for in-hospital mortality and to develop and internally validate a combined prediction model based on readily accessible clinical and laboratory indicators, with the goal of enabling risk stratification to guide clinical management.

## Methods

### Ethics statement

This study was approved by the Institutional Review Board of Hainan General Hospital (approval number: EC-YLY-2025-106-01). Written informed consent was waived due to the retrospective nature of the study, and all data were anonymized prior to analysis.

### Study design and population

This retrospective cohort study was conducted at Hainan General Hospital, a tertiary care center in a melioidosis-endemic region of China. Clinical data of patients admitted with a diagnosis of melioidosis between June 2010 and June 2024 were reviewed.

### Inclusion and exclusion criteria

Patients were included if they met the following criteria: (1) diagnosis of melioidosis based on compatible clinical presentation in an endemic area and confirmed by culture or whole-genome sequencing of *B. pseudomallei*; (2) availability of complete key clinical data. Exclusion criteria were: (1) co-infection with other active systemic infections (e.g., HIV, active tuberculosis); (2) missing essential laboratory data or outcome information; (3) death or discharge within 24 hours of admission (to minimize bias from extremely short stays). A total of 283 patients were included and categorized into survival (n = 235) and in-hospital death (n = 48) groups based on their clinical outcome.

### Data collection

Data were extracted from the hospital’s electronic medical record system, including:

**Demographics and history:** age, sex, occupation (farmer), smoking history, alcohol use.

**Clinical features:** body temperature on admission, total length of hospital stay (days from admission to discharge or death), presence of sepsis (as defined by Sepsis-3 criteria), and types of focal infections (pulmonary, localized abscess, urinary tract).

**Comorbidities:** hypertension, diabetes mellitus, chronic liver disease/cirrhosis, chronic kidney disease, malignancy, immune dysfunction, hypoalbuminemia, malnutrition, anemia.

**Laboratory indicators:** Two sets of laboratory parameters were collected: (1) initial values within 24 hours of admission, including white blood cell count (WBC), hemoglobin, and erythrocyte sedimentation rate (ESR); and (2) the last available measurements during hospitalization (i.e., the final value recorded prior to discharge in survivors or prior to death in non-survivors), including neutrophil percentage (NEUT%), platelet count (PLT), C-reactive protein (CRP), urea nitrogen, blood glucose, and glycosylated hemoglobin (HbA1c). Data on interleukin-6 (IL-6) and procalcitonin (PCT) were also collected but were incomplete.

### Statistical analysis

#### Univariate analysis and data description.

Statistical analysis was performed using SPSS software (version 26.0). Continuous variables were tested for normality using the Kolmogorov-Smirnov test and are presented as median with interquartile range (IQR). Group comparisons were made using the Mann-Whitney U test. Categorical variables are presented as frequencies (percentages) and were compared using the Chi-square test or Fisher’s exact test, as appropriate.

#### Multivariate analysis.

Variables with a univariate *P* value < 0.05 were entered into a binary logistic regression model (forward LR method) to identify independent risk factors. Variables with >50% missing data (IL-6, PCT) were excluded from the multivariate analysis and were not presented in the univariate comparisons due to the potential for bias. With 48 mortality events and four predictors in the final model, the events-per-variable ratio was 12, exceeding the recommended minimum of 10. Multicollinearity among the four predictors was assessed using the variance inflation factor (VIF); all VIF values were below 5, indicating no significant collinearity.

#### Model evaluation and internal validation.

The predictive performance of individual variables and the combined logistic regression model was evaluated using receiver operating characteristic (ROC) curve analysis. The DeLong test was used to compare the area under the curve (AUC) values. The optimal cutoff point was determined by maximizing the Youden index. Internal validation of the final model was performed using bootstrap resampling with 1,000 replicates to calculate optimism-corrected performance estimates. Model calibration was assessed using the Hosmer-Lemeshow goodness-of-fit test and a calibration plot. A two-tailed P value < 0.05 was considered statistically significant.

## Results

### Baseline characteristics and univariate analysis

The baseline characteristics, clinical features, and underlying comorbidities of the 283 patients are summarized in Table 1. There were no significant differences between survivors and non-survivors in terms of sex, age, occupation (farmer), or body temperature on admission. However, non-survivors had significantly lower rates of smoking and alcohol use (both *P* < 0.05). In terms of clinical features, non-survivors had a significantly shorter median hospital stay (3.5 vs. 21.0 days, *P* < 0.001) and a higher incidence of sepsis (81.3% vs. 60.9%, *P* = 0.007). Non‑survivors were also significantly more likely to be admitted to the ICU than survivors (56.3% vs. 11.1%, χ² = 53.37, *P* < 0.001). The prevalence of most underlying comorbidities, including diabetes mellitus, was similar between the two groups. Although hospital stay was significantly shorter in non-survivors, it was not included in the prediction model due to inherent time-dependent bias (i.e., length of stay is itself determined by survival status).

**Table 1:** Baseline characteristics, clinical features, and underlying comorbidities of melioidosis patients, stratified by in-hospital outcome.

Indicators	Survival (n = 235)	Death (n = 48)	Statistic	*P* value
Demographics				
Male, n(%)	208 (88.5)	41 (85.4)	χ² = 0.361	0.548
Age, years, Median (IQR)	52.0 (43.0, 61.0)	53.5 (48.0, 63.0)	Z = -1.346	0.178
Farmer, n(%)	92 (39.2)	16 (33.3)	χ² = 0.571	0.450
Smoking, n(%)	129 (54.9)	17 (35.4)	χ² = 6.054	0.014
Drinking, n(%)	94 (40.0)	11 (22.9)	χ² = 4.985	0.026
Clinical Features and Complications				
Body temperature, Median (IQR), °C	38.3 (36.8, 39.5)	38.7 (37.5, 40.0)	Z = -1.399	0.162
Hospital Stay, days, Median (IQR)	21.0 (13.0, 30.3)	3.5 (1.0, 8.0)	Z = -9.080	<0.001
Sepsis, n(%)	143 (60.9)	39 (81.3)	χ² = 7.381	0.007
Pulmonary infection, n(%)	149 (63.4)	33 (68.8)	χ² = 0.496	0.481
Local abscess, n(%)	82 (34.9)	10 (20.8)	χ² = 3.591	0.058
Urinary tract infection, n(%)	24 (10.2)	6 (12.5)	χ² = 0.220	0.639
ICU admission, n(%)	26 (11.1)	27 (56.3)	χ² = 53.469	<0.001
Underlying Diseases				
Any, n(%)	205 (87.2)	43 (89.6)	χ² = 0.203	0.652
Diabetes, n(%)	153 (65.1)	36 (75.0)	χ² = 1.763	0.185
Hypertension, n(%)	45 (19.1)	12 (25.0)	χ² = 0.848	0.357
Chronic liver disease/Cirrhosis, n(%)	53 (22.6)	8 (16.7)	χ² = 0.817	0.366
Tumor, n(%)	20 (8.5)	4 (8.3)	Fisher’s exact	1.000
Immunodeficiency, n(%)	14 (6.0)	0 (0.0)	Fisher’s exact	0.171
Chronic kidney disease, n(%)	37 (15.7)	9 (18.8)	χ² = 0.264	0.607
Hypoproteinemia, n(%)	84 (35.7)	16 (33.3)	χ² = 0.101	0.750
Malnutrition, n(%)	16 (6.8)	7 (14.6)	χ² = 2.270	0.132
Anemia, n(%)	34 (14.5)	8 (16.7)	χ² = 0.150	0.696

Data are presented as n (%) or median (interquartile range, IQR). Categorical variables were compared using the Chi-square test, corrected Chi-square test, or Fisher’s exact test, as appropriate. Continuous variables were compared using the Mann-Whitney U test.

### Comparison of laboratory parameters

As shown in Table 2, based on the last available measurements during hospitalization, non-survivors had significantly higher neutrophil percentage, CRP, urea nitrogen, IL-6, and PCT levels, alongside a significantly lower platelet count (all *P* < 0.001). No significant differences were observed in fasting blood glucose, HbA1c, WBC, hemoglobin, or ESR. Due to >50% missing data, IL-6 and PCT were not included in further multivariate analysis.

**Table 2:** Comparison of laboratory parameters between survival and death groups (last available measurements during hospitalization).

Indicators	Survival (n = 235)	Death (n = 48)	Z value	*P* value
Platelet count (PLT, × 10⁹/L)	300.5 (211.5, 397.5)	58.0 (19.0, 138.8)	-8.579	<0.001
Neutrophil percentage (NEUT%, last measurement)	66.00 (52.85, 76.45)	86.70 (82.57, 92.00)	-7.234	<0.001
C-reactive protein (CRP, mg/L)	126.2 (51.6, 207.6)	246.0 (166.8, 305.7)	-5.685	<0.001
Urea nitrogen (mmol/L)	4.30 (3.06, 6.11)	10.06 (4.89, 16.20)	-5.985	<0.001
IL-6*	88.82 (37.95, 241.30)	5000.00(362.00, 5000.00)	-3.792	<0.001
Fasting blood glucose(mmol/L)	9.04 (5.35, 14.75)	9.53 (7.50, 14.45)	-1.796	0.073
HbA1C(%)	10.80 (8.38, 12.70)	10.95 (8.55, 12.33)	-0.047	0.962
White blood cell count (WBC, last measurement)	7.79 (6.01, 10.51)	7.70 (4.80, 11.98)	-0.136	0.892
Hemoglobin (g/L, last measurement)	117.00 (100.50, 132.00)	115.00 (94.00, 133.00)	-0.429	0.668
ESR(mm/h)	77.00 (51.00, 106.00)	64.50 (43.75, 103.00)	-1.010	0.312

Data for IL-6 and procalcitonin (PCT) were incomplete (>50% missing) and thus were not included in the multivariate analysis. Reference ranges: platelet count 125–350 × 10^9^/L, neutrophil percentage 40–75%, CRP < 10 mg/L, urea nitrogen 2.9-8.2 mmol/L.

### Multivariate analysis and prediction model development

Variables significant in univariate analysis (excluding IL-6 and PCT) were entered into the logistic regression model. Four independent risk factors for in-hospital mortality were identified (Table 3): higher last available neutrophil percentage (OR = 1.040, 95% CI: 1.007-1.074), lower last available platelet count (OR = 0.987, 95% CI: 0.982-0.993), higher CRP level (OR = 1.008, 95% CI: 1.002-1.013), and higher urea nitrogen level (OR = 1.117, 95% CI: 1.032-1.209).

**Table 3:** Multivariate logistic regression analysis of independent predictors for in-hospital mortality.

Variable	*β*	SE	Wald χ²	*P* value	OR (95% CI)
Neutrophil percentage (last measurement)	0.040	0.016	5.836	0.016	1.040 (1.007–1.074)
Platelet count (last measurement)	-0.013	0.003	19.918	<0.001	0.987 (0.982–0.993)
CRP	0.008	0.003	6.543	0.011	1.008 (1.002–1.013)
Urea nitrogen	0.110	0.040	7.444	0.006	1.117 (1.032–1.209)
Constant	-4.451	1.583	7.912	0.005	0.012

The model was constructed using forward stepwise logistic regression (Likelihood Ratio). Variable assignment: outcome (death = 1, survival = 0); all continuous variables entered as original values.

### Performance of the prediction model

**Fig 1:**
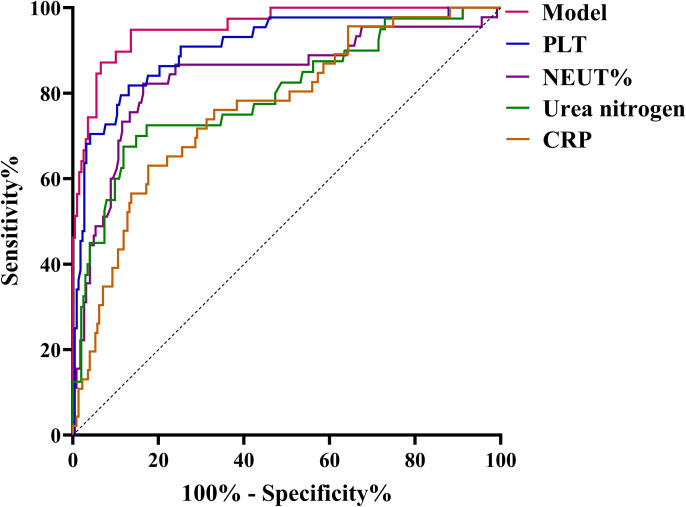
Receiver operating characteristic (ROC) curves of the combined prediction model and individual indicators for predicting in-hospital mortality. Note: The combined model includes neutrophil percentage, platelet count, CRP, and urea nitrogen. AUC values are presented in Table 4.

ROC curve analysis demonstrated that the combined model incorporating all four independent risk factors achieved outstanding discriminative ability, with an AUC of 0.957 (95% CI: 0.925-0.989) (Table 4, Fig 1). At the optimal predicted probability cutoff, the model yielded a sensitivity of 94.9% and a specificity of 86.4%. The AUC of the combined model was significantly higher than that of any single variable (all *P* < 0.001).

**Table 4:** Performance comparison of individual predictors and the combined model for predicting in-hospital mortality.

Indicator	Cut-off	AUC (95% CI)	Sensitivity	Specificity	Youden Index
Combined model	–	0.957 (0.925-0.989)	0.949	0.864	0.813
Platelet count (last measurement)	≤77.5	0.910 (0.854-0.967)	0.718	0.975	0.693
Neutrophil percentage (last measurement)	>80.45	0.842 (0.767-0.917)	0.822	0.836	0.658
Urea nitrogen	>7.905	0.811 (0.728-0.894)	0.692	0.884	0.576
CRP	>223.93	0.742 (0.654-0.830)	0.641	0.803	0.444

AUC, area under the curve; CI, confidence interval. The combined model was derived from the logistic regression equation including neutrophil percentage, platelet count, CRP, and urea nitrogen. Cutoff values for individual indicators were determined by maximizing the Youden index from the ROC analysis.

### Internal validation of the prediction model

**Fig 2:**
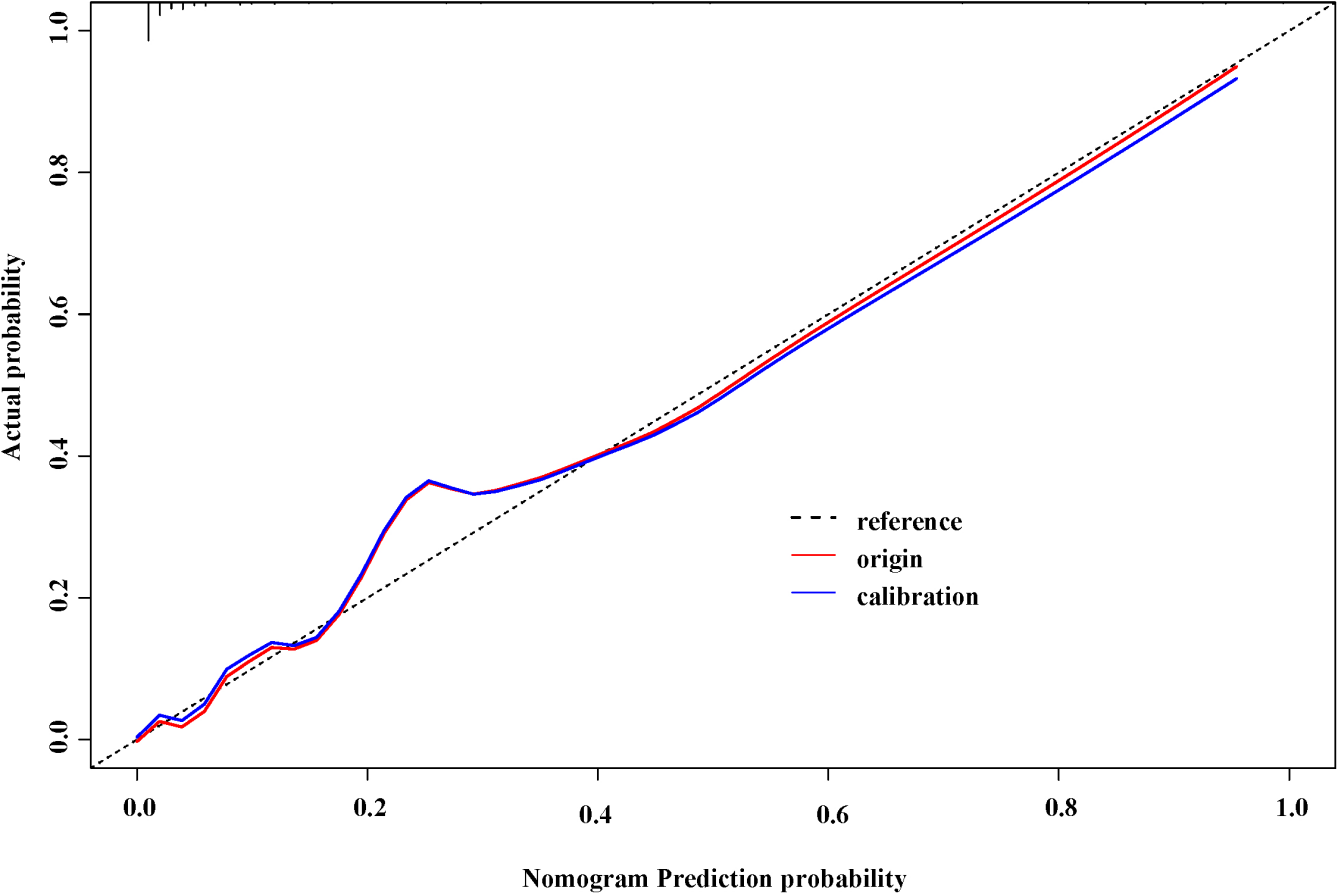
Calibration curve of the logistic regression model for predicting in-hospital mortality. Note: The diagonal line represents ideal calibration (perfect prediction). The apparent line shows the model’s performance on the original data, and the bias-corrected line represents the performance after bootstrap resampling (1,000 replicates). Close alignment between the bias-corrected line and the ideal line indicates good calibration.

Internal validation was performed using bootstrap resampling with 1,000 replicates. The Hosmer-Lemeshow goodness-of-fit test indicated good calibration, with no significant difference between observed and predicted mortality (χ² = 3.015, df = 8, *P* = 0.933). The calibration plot showed high agreement between the predicted probabilities and actual outcomes (Fig 2).

### Time from last available measurement to death

Among the 48 non-survivors with complete data for all four model parameters, we calculated the time interval between the latest of the last available measurements (i.e., the most recent complete blood count, CRP, and urea nitrogen) and the date of death. The median interval was 1.0 day (IQR: 0.5-2.0 days; range: 0–7 days). In 27.1% of cases, all four parameters were available on the calendar day of death; in the remaining 72.9%, the last measurement occurred at least 12 hours before death (using either the recorded time of death or, when only the date was available, assuming death at 00:00 on that date). This indicates that the model could provide a clinically meaningful lead time of approximately 1 day before death in a typical patient.

## Discussion

In this retrospective study from a major melioidosis-endemic center in China, we identified a concise set of four independent predictors of in-hospital mortality based on the last available measurements during hospitalization: elevated neutrophil percentage, thrombocytopenia, and elevated CRP and urea nitrogen. A model combining these routinely available parameters exhibited outstanding predictive accuracy (AUC 0.957) with excellent calibration after internal validation.

The identified biomarkers have strong pathophysiological links to severe infection. Thrombocytopenia in melioidosis is associated with disseminated intravascular coagulation and endothelial activation, as evidenced by related coagulation abnormalities [12]. Elevated neutrophil percentage is a well-established indicator of systemic inflammation and bacterial infection severity [13]. However, the prognostic significance of neutrophil count in melioidosis may be complex, as one study reported that a low leukocyte, neutrophil, and lymphocyte count (“tri-low phenotype”) was associated with early mortality in this disease [14]. Elevated CRP, an acute-phase reactant, further corroborates the intense inflammatory state [15], while elevated urea nitrogen signals acute kidney injury, a well-established poor prognostic marker in sepsis and melioidosis [16,17]. The model’s capture of these key sepsis pathways (inflammation, coagulopathy, organ dysfunction) likely explains why the clinical syndrome of sepsis was superseded by its objective laboratory correlates. We note that the platelet cut‑off (≤77.5 × 10⁹/L) is lower than in some other studies, which likely reflects the peri‑mortem timing of measurements in non‑survivors.

Notably, while diabetes is a key risk factor for contracting melioidosis [5,6,18], it did not retain independent significance for in-hospital mortality in our multivariate model. This finding is consistent with some contemporary studies suggesting that in acute sepsis, the severity of the infection may eclipse the prognostic influence of underlying diabetes [8,19]. Similarly, a multicenter study from the same region in Hainan also reported that diabetes was not an independent predictor of mortality in melioidosis patients [20]. Our cohort’s high baseline diabetes prevalence (67.8%) may have also diluted its discriminatory power.

The principal strength of our model lies in its high performance using only inexpensive, universal, and routinely collected parameters: the last available complete blood count and basic metabolic panel during hospitalization. This makes it inherently suited for resource-limited endemic settings, a core focus of this journal. In practical terms, this model requires only a complete blood count and basic metabolic panel. These tests are available even in district hospitals in melioidosis-endemic regions. A simple bedside score derived from the regression coefficients could be calculated within minutes, enabling identification of high-risk patients and resource allocation. For clinical translation, this model could be operationalized in this way or integrated into electronic health records to automatically flag patients at high risk of mortality, prompting intensified monitoring and treatment.

### Clinical implications of the lead time

Our analysis showed a median lead time of 1 day before death. Even a lead time of 12–24 hours can be sufficient to escalate care in a hospitalized patient [21,22]. For melioidosis, where deterioration can occur rapidly, this window could allow clinicians to reassess antibiotic coverage or consider intensive care support, although the impact of such interventions in the peri‑mortem period requires further investigation.

### ICU admission and model performance

As shown in Table 1, non‑survivors were significantly more likely to be admitted to the ICU than survivors (56.3% vs. 11.1%, χ² = 53.37, *P* < 0.001), reflecting their greater illness severity. However, ICU admission alone does not ensure survival; despite ICU care, 27 patients died. Our model provided additional risk stratification beyond ICU status, identifying patients at extremely high risk of death even within the ICU setting. This suggests that the model could complement clinical judgment by flagging patients who may need further escalation of care despite ICU admission.

### Implementation considerations

To facilitate clinical adoption, we propose a simplified risk score based on the regression coefficients. For instance, assigning points to each parameter at its optimal cutoff (e.g., platelet count ≤77.5 × 10⁹/L, neutrophil percentage >80.45%, CRP > 223.93 mg/L, urea nitrogen >7.905 mmol/L) yields a total score that can be rapidly calculated at the bedside. Patients with high scores would be flagged for intensive care referral or early escalation of antibiotics. We note that this simplified score requires external validation before clinical application.

### Limitations

Our study has several limitations. Its retrospective, single-center design may introduce selection bias and limits control for unmeasured confounders such as detailed treatment timelines. Additionally, detailed data on antibiotic regimens and time to appropriate therapy were not available, which may influence outcomes. Specifically, we could not determine the proportion of patients receiving effective *B. pseudomallei* therapy at the time of the last available measurements, because antibiotic timing and susceptibility data were not systematically captured for all patients. The use of last available laboratory measurements rather than admission values introduces inherent time-dependent bias (i.e., measurements taken closer to death in non‑survivors versus at discharge in survivors). However, this approach captures cumulative physiological deterioration, and we acknowledge that the model is intended for risk assessment during hospitalization rather than exclusively at admission. Important emerging biomarkers (IL-6, PCT) could not be analyzed due to high missing data rates. Although internal validation using bootstrap resampling showed good performance, external validation in prospective, multi-center cohorts from diverse epidemiological settings is essential to confirm generalizability. The sample size, particularly of the non-survivor group (n = 48), while adequate for the primary analysis, may limit precision in subgroup analyses. We did not evaluate the model’s performance using admission values; therefore, we cannot compare its predictive ability with that of peri-mortem measurements. Future studies should assess whether the same four parameters measured at admission can achieve similar discrimination. Because we used last-available measurements (pre-death in non-survivors vs. pre-discharge in survivors), the model is not suitable for admission-time risk stratification. It should be seen as a descriptive or monitoring tool for in-hospital mortality risk.

## Conclusion

We developed and internally validated a high-accuracy prediction model for in-hospital mortality in melioidosis using four routine clinical indicators based on the last available measurements during hospitalization. This practical tool holds significant promise for risk stratification during hospitalization, particularly in resource-conscious endemic regions. Implementing such a model could enhance clinical decision-making, optimize resource allocation, and potentially improve outcomes through the timely identification of high-risk patients.

## Supporting information

S1 Table De-identified minimal dataset underlying the study results. (XLSX)
